# Time to recovery and its predictors among under-five children admitted with severe pneumonia in East Wallaga Zone public hospitals, western Ethiopia, 2023; a retrospective cohort study

**DOI:** 10.1186/s12887-024-04937-2

**Published:** 2024-07-18

**Authors:** Imana Raga Dinka, Dejene Seyoum, Sidise Debelo, Gudetu Fikadu, Misganu Teshoma Regasa, Hunde Fayera Abdena, Roba Tolessa Jiren, Worku Garuma Ayana

**Affiliations:** 1 Department of Public Health, Department of Midwifery, Institute of Health Sciences, Wallaga University, Nekemte, Oromia Ethiopia; 2 Department of Midwifery, Institute of Health Sciences, Wallaga University, Nekemte, Oromia Ethiopia; 3 Nekemte Health Science College, Oromia National Health Bureau, Nekemte, Oromia Ethiopia

**Keywords:** Time to recovery, Severe pneumonia, Predictors, Under-five children

## Abstract

**Background:**

Globally, pneumonia is one of the leading causes of morbidity and mortality as well as hospitalization burden for under-five children. Despite significant initiatives implemented to reduce morbidity and mortality from pneumonia in under-five children, little is known regarding the time to recovery and its predictors among under-five children admitted with severe pneumonia in Ethiopia. Hence, this study intended to estimate the median time to recovery and its predictors among under-five children admitted with severe pneumonia in East Wallaga zone public hospitals, western Ethiopia; 2023.

**Methods:**

An institution-based retrospective cohort study was conducted among 383 under-five children who were admitted with severe pneumonia in East Wallaga zone public hospitals from January 2017 to December 2022. A systematic sampling method was used to select eligible medical records. EpiData Version 4.6 was used to enter the data and analyzed using STATA Version 17.0. Cox-proportional hazard assumption test and model fitness were checked. Variables with P-value ˂ 0.25 at bivariable Cox regression analysis were selected for the multivariable Cox proportional model. A multivariable Cox regression model with 95% CI and Adjusted Hazard Ratio (AHR) was used to identify a significant predictor of time to recovery from severe pneumonia at a P-value < 0.05.

**Results:**

At the end of the follow-up, 356 observations were developed an event (recovered) with the median time to recovery of 4 days with IQR of 3–5 days. The overall incidence rate of recovery was 22.26 per 100 (95% CI: 20.07–24.70) person-days observations. Being rural residency (AHR: 0.75, 95% CI: 0.60–0.93), late presenters for seeking care (AHR = 0.70, 95% CI: 0.53–0.93), presence of danger sign at admission (AHR = 1.46, 95% CI: 1.15–1.83), and presence of comorbidity (AHR = 1.63, 95% CI, 1.31–2.04) were found to have a statistically significant association with prolonged recovery time.

**Conclusion:**

The median time to recovery from severe pneumonia was long, and factors such as Residence, co-morbidity, presence of danger signs, and duration prior to seeking care were statistically significant predictors of recovery time from severe pneumonia. Hence, due attention has to be given to increasing the community’s health-seeking behavior to visit health facility early and especial attention should be given for children with danger signs and comorbidity.

**Supplementary Information:**

The online version contains supplementary material available at 10.1186/s12887-024-04937-2.

## Background

Pneumonia is defined as an inflammation of the parenchymal tissues of the lung, such as the alveoli and the bronchioles [1]. The World Health Organization (WHO) has defined pneumonia in children clinically based on either a cough, difficulty breathing, rapid respiratory rate, chest indrawing, or a decreased level of consciousness [2].

Pneumonia can be caused by bacteria, viruses, and fungi. Streptococcus pneumoniae is the most common cause of bacterial pneumonia in children, followed by Haemophilus influenzae type b [3]. Respiratory syncytial virus is the most common viral cause of pneumonia [4].

The WHO guidelines classified the respiratory symptoms of under-five children into four categories. These are: Children with cough and cold who did not have signs of pneumonia were classified as no pneumonia, children with fast breathing were classified as pneumonia, and Children who had chest indrawing with or without fast breathing were classified as severe pneumonia, Children who had any general danger signs were classified as severe pneumonia or very severe disease [5, 6].

Globally, pneumonia is one of the leading causes of morbidity and mortality, as well as hospitalization burden, for under-five children [7]. In 2020, the United Nations Children‘s Fund (UNICEF) reported that pneumonia is the world’s leading infectious killer of children, claiming the lives of more than 800,000 children under the age of five every year, more than 2,000 every day [8]. It is also a common reason for children to be admitted to hospitals, especially in resource-poor communities [9]. Africa loses thousands of children due to pneumonia each year. It causes around 750,000 child deaths per year in sub-Saharan African countries [10]. A recent WHO estimation suggests that pneumonia is most abundant in South Asia, India, Pakistan, China, and sub-Saharan Africa, including Ethiopia. In Ethiopia, 90% of the deaths of children under five years of age are caused by pneumonia, diarrhea, malaria, measles, and problems of the newborn, and pneumonia is the preceding one among them [10, 11].

Around the world, significant efforts have been made to minimize pneumonia-related morbidity and mortality [12]. However, the main difficulty is the increasing duration of stay in the hospital and the poor response to antibiotic treatments, which has resulted in a decrease in pneumonia patient survival [13]. A longer period of hospitalization is known to raise the risk of local and systemic infection, which can lead to complicated pneumonia, which is characterized by severe illness and a complicated disease condition by causing one or more of the following: Para pneumonic effusion, empyema, necrotizing pneumonia, and lung abscess [14]. It also has huge economic disadvantages, either directly through medical expenses or indirectly through the loss of working hours suffered by parents of sick children. These negative impacts of inpatient management of severe pneumonia become more pronounced if the length of stay is prolonged and the time of recovery is unpredicted [7, 15].

The average length of stay in hospitals is considered a measure of efficiency. According to the US Centers for Disease Control and Prevention, the average length of hospital stay for children under 15 years (excluding neonates) admitted with severe pneumonia is five days. Any hospital stay that lasts more than five days is considered to be prolonged [15]. A study conducted on the predictors of duration and treatment failure of severe pneumonia in Nepal estimates that the median time till recovery was 49 h [16]. Another retrospective follow-up study conducted in Gondor, Ethiopia, showed that the median time to recovery from severe pneumonia was 3 days [17].

Evidence showed that; the management of severe pneumonia requires early identification, appropriate referral, and availability of good quality care [15]. WHO also reviewed literature intending to develop a simplified approach that could increase the number of children receiving the correct treatment for pneumonia that in return fasten their recovery time. The management includes advice on home therapy, antibiotics for home treatment, and referral to a higher-level health care center [6].

Despite the programs and techniques indicated above, pneumonia is still one of the leading causes of morbidity and mortality in children worldwide and in developing countries, including Ethiopia [18], which still needs more detailed, valid, and reliable information. Though some scientific researches have been published on the time to recovery from severe pneumonia and its predictors among under-five children, they were mainly limited to one specialized hospital, and the median recovery time in these studies was 3–4 days. These studies also reported that; Lack of exclusive breastfeeding, Age group, comorbidity, Presence of danger signs at admission, Residence, Stunting, Vaccination status, and duration prior to seeking care as significant predictors of time to recovery from severe pneumonia [15, 16, 19–22]. There are only limited studies in Ethiopia that report the estimated time of recovery and predictors of severe pneumonia among under-five children. Especially in western Ethiopia, particularly in the study area, no similar study was conducted regarding recovery time from severe pneumonia and its predictors among under-five children. Hence, this study aimed to estimate the median time to recovery and its predictors among under-five children admitted with severe pneumonia in East Wallaga zone public hospitals, western Ethiopia.

## Materials and methods

### Study design, study area, and period

An institutional-based retrospective cohort study was conducted in East Wallaga Zone public hospitals from January 2017 to December 2022. Chart review was performed from January 10, 2023, to February 10, 2023. East Wallaga is one of the 21 zones in the Oromia region and is located in western Ethiopia, 328 km from Ethiopia’s capital city, Addis Ababa (Finfine). It is climatically categorized as “Woyna Dega,” and the rainfall is about seven months, nearly from March to September in the year.

East Wallaga zone is bordered in the east by the West Showa zone, in the north by the Horo Guduru Wallaga zone and Benishangul Gumuz, in the south by the Buno Bedele zone, and in the west by the West Wallaga zone. East Wallaga is divided into 17 woredas, 327 kebeles, and one town. There are five government hospitals (Nekemte Specialized Hospital, Wallaga University Referral Hospital, Arjo Primary Hospital, Sire Primary Hospital, and Gida Ayana General Hospital), 65 health centers, and 395 health posts in the East Wallaga zone. Its population is estimated to be 1,697,638 people, which includes 831,843 males and 865,795 females in the year 2016/17 which is projected from the 2007 Ethiopia Central Statistical Agency.

The study was conducted at Nekemte Comprehensive Specialized Hospital, Wallaga University Referral Hospital, and Gida Ayana General Hospital. According to the hospital’s monthly report, 1420, 814, and 583 under-five children were admitted with severe pneumonia from January 2017 to December 2022 in Nekemte specialized hospital, Wallaga University Referral Hospital, and Gida Ayana General Hospital, respectively.

### Populations

The source populations were all the under-five children with severe pneumonia admitted in East Wallaga Zone public hospitals, and the study population was all under-five children with severe pneumonia admitted in selected East Wallaga Zone public hospitals during the study period (from January 2017 to December 2022).

### Inclusion and exclusion criteria

All children aged 2–59 months with severe pneumonia who were admitted in selected East Wallaga Zone public hospitals from January 2017 to December 2022 were included in the study. Lost medical records of children with severe pneumonia that weren’t available at the time of data collection were excluded from the study.

### Sample size determination

The sample size was determined for common predictors that are found to be significantly associated with time-to-recovery from severe pneumonia among under-five children, according to studies in the different areas [17, 21, 22].

All the possible sample sizes were calculated, and the largest sample size (*n* = 348) was selected from the retrospective cohort study conducted in Gonder, North Ethiopia, by considering duration prior to seeking care as a major predictor using the Freedman method of sample size determination [23].

After adding 10% to the sample size in order to account for incomplete records, the final sample size of this study was **383**.

The total sample size was calculated as:

$$\:n=\frac{E}{Pr\left(E\right)}$$

Where: n = total sample size needed

E = the number of events that interested.

Pr(E) = the probability that the event of interest (which is a recovery in this context) occurs.

$$\:\text{E}=\frac{{(\text{Z}\frac{\alpha\:}{2}+\text{Z}{\upbeta\:})}^{2}}{{\left(lnAHR\right)}^{2}\:P(1-P)}$$

Where: E = number of events that interested.

Zα/2: Z value at 95% confidence interval = 1.96.

Zβ: the power of the study = 0.84.

P: Recovery rate.

AHR: Adjusted Hazard Ratio.

### Sampling technique

First, by using simple random sampling (the lottery method), three hospitals were selected out of five public hospitals found in the East Wallaga Zone. The selected hospitals were Wallaga University Referral Hospital, Nekemte Specialized Hospital, and Gida Ayana General Hospital. For record reviews, five consecutive years (from January 2017 to December 2022) were chosen deliberately. Starting from the recent month backward, based on the sequence of their card numbers, a systematic sampling procedure was used to select an adequate number of samples (patient charts) until the required sample size was obtained. During the five years, the total number of under-five children admitted with severe pneumonia was two thousand eight hundred seventeen (2817). By calculating the interval from the sampling frame, the total sample size for each year was proportionally allocated (*N* = 2817), with sample size *n* = 383, which gave a sampling interval (K) of 7. Then, using the patients’ record order, which was listed in the log book, study participants (charts) were selected at every 7-number interval until reaching the total sample size. The first participant (chart) was selected by the lottery method of sampling.

### Data collection tools and data collection procedures

Data were collected using a pre-tested and structured checklist. The data collection format was adapted after a detailed review of similar literature and observing under-five patient charts with severe pneumonia and pediatric ward registration log book to establish the trend of registration, physical examination, and history taking. The data collection tool was designed by considering study variables such as sociodemographic, nutritional, and clinical predictors from medical records.

All available information on patient records was checked after appropriate data.

extraction format was prepared in English to extract all the relevant variables.

to meet the study objectives from patient charts. The time from the diagnosis of severe pneumonia was the starting point for retrospective follow-up and the endpoint was the date of recovery, the date of loss to follow-up, or the date of death. All charts of under-five children with severe pneumonia admitted in selected East Wallaga zone Public Hospitals (Wallaga University Referral Hospital, Nekemte Specialized Hospital, and Gida Ayana General Hospital) from January 2017 to December 2022 were retrieved from the HMIS registration book. The records of all study participants were selected according to the eligibility criteria. Recovery was confirmed by discharge note complemented by registration and was identified by their medical record number. One BSc Nurse for supervision and three BSc Nurses for data collection were recruited. One-day training was given to data collectors and a supervisor regarding the significance of the study and ways of the data collection process. The supervisor has monitored the data collection process.

### Data quality control

To ensure the quality of the data, a supervisor and data collectors were trained on how and what information they should collect from the targeted data sources. Prior to data collection, the data extraction form was tested by taking 5% of randomly selected patient charts from Wallaga University Referral Hospital to assess the reliability and consistency of the data collection tool.

### Study variables

#### Dependent variable

Time to recovery from severe pneumonia.

### Independent variables

- Socio-demographic factors (age, sex, residence).
- Presence of co-morbid diseases (SAM, HAAD, CHD, Rickets, Pertussis, Down syndrome, and others).
- Nutritional status of children (stunting, wasting, underweight, exclusive breastfeeding).
- Clinical characteristics and danger signs (head nodding, impaired consciousness, hypoxia, vomiting everything, abnormal body movement, fever).
- Duration prior to seeking care (early and late presentation).
- Vaccination status of children (fully vaccinated, partially vaccinated, unvaccinated, unknown).
- Drug regimen (crystalline penicillin, ceftriaxone, ampicillin & gentamycin).

### Operational definitions

#### Recovery

Children improved from severe pneumonia and were discharged from the hospital according to the criteria of IMCI (when respiratory distress has resolved, there is no hypoxemia (oxygen saturation ≥ 90%), feeding well, can take oral medication, and their parents understand the signs of pneumonia) [24].

#### Event

refers to recovery from an illness (severe pneumonia) during the study period [15].

#### Survival time

is the time starting from the date of admission to recovery from severe pneumonia [17].

#### Censored

refers to children referred, died, absconded, Left against medical advice, or discharged for any reason without recovery during the study period [16].

#### Danger sign

The child having loss of consciousness, abnormal body movement, vomiting everything, convulsions, and inability to feed in addition to severe pneumonia [25].

#### Co-morbidity

Any disease condition (acute or chronic) present at admission in addition to severe pneumonia which includes hyperactive airway disease (childhood asthma), severe acute malnutrition, congenital heart disease, congestive heart failure, rickets, down syndrome, retroviral infection, tuberculosis, acute gastroenteritis, pertussis, anemia, meningitis, measles, bronchitis, heart failure and others [21].

#### Exclusive breastfeeding

Children who are introducing only breast milk except for ORS, drops, and syrups (vitamins, medicine, and minerals) with his/her age during the age of less than 6 months [17].

#### Vaccination status

Full vaccination status is defined as children who had completed all forms of vaccinations; partially vaccinated (children who had taken at least one dose of PCV) and non-vaccinated (children who had never been vaccinated PCV and other vaccines) [26].

#### Severe pneumonia

A child with cough or difficulty of breath and fast breathing plus any general danger sign [6].

#### Time to seeking care

Children getting care within 5 days after getting sick are considered as early presenters while those children getting care after 5 days are considered as late presenters [21].

### Data processing and analysis

Data were entered into EpiData version 4.6 and then exported to STATA version 17 for further analysis. Before analysis, data were cleaned and edited by using simple frequencies and cross tabulation; re-categorization of categorical variables and categorization of continuous variables was done to be suitable for analysis. Descriptive non-parametric survival analysis such as Kaplan Meier survival curve and log rank were used to test any difference in survival probability in categorical covariates. Days were used as a time scale to calculate the median time to recovery. A z-score chart from the WHO guideline was used for the assessment of the nutritional status of study participants.

A Cox proportional hazard regression model was used to determine factors associated with recovery time. Before running the Cox proportional hazard regression model, multicollinearity was checked using the Variance Inflation Factor (VIF) [27], and no problem of multicollinearity was seen in this study. Factors associated with recovery time at p-value ˂ 0.25 in bivariable Cox regression were selected for multivariable Cox regression analysis. Moreover, further variable selection was undertaken using a stepwise backward variable selection approach. Adjusted Hazard Ratios (AHR) with 95% confidence intervals were computed and statistical significance was declared when it is significant at a 5% level (p-value *<* 0.05). To assess model adequacy for the proportional hazard model, the proportional hazard assumption was checked by log-log plot and global test, and overall model adequacy of the proportional hazard model was assessed by using the Cox Snell residual graph. Finally, results were compiled and presented using tables, graphs, and texts.

## Results

### Socio-demographic characteristics of study participants

Out of the total 376 children in the retrospective cohort study; females accounted for 190(50.53%). The median age of the children was 12 months (IQR = 5–24 months), with 48.94% of the children aged 2 to 11 months and 35.9% aged 12 to 35 months (Table 1).

**Table 1:** Socio-demographic characteristics of under-five children admitted with severe pneumonia in East Wallaga Zone Public hospitals, western Ethiopia, January 2017 to December 2022

Variables	Category	Survival status		Total (*n* = 376)
		Recovered	Censored	
		No	No	No (%)
Age	2–11 months	175	8	183(48.67)
	12–35 months	127	7	134(35.64)
	36–59 months	54	5	59(15.69)
Sex	Male	177	9	186(49.47)
	Female	179	11	190(50.53)
Residence	Urban	143	5	148(39.36)
	Rural	213	15	228(60.64)

### Nutritional and vaccination status of study participants

For the first six months, 281(74.73%) of the children were exclusively breastfed and 62(16.49%) of them were unknown. To classify children’s nutritional status, anthropometric measurements were compared to WHO child growth standards. From the overall study participants, 40(10.64%), 17(4.52%), and 31(8.24%) of them were categorized as underweight, stunting, and wasting respectively. Regarding vaccination status 104(27.66%) of the children were fully vaccinated, 173(46.01%) were up‑to‑date (vaccinated according to their age), 41(10.90%) were partially vaccinated and 31(8.24%) were not vaccinated at all according to the National Expanded Program of Immunization (EPI) (Table 2).

**Table 2:** Nutritional and vaccination status among under-five children admitted with severe pneumonia in East Wallaga Zone public hospitals, Western Ethiopia, January 2017 to December 2022

Variables	Category	Survival status		Total (*n* = 376)
		Recovered	Censored	
		N*o*	N*o*	N*o* (%)
Exclusive breast feeding	Yes	271	10	281(74.73)
	No	31	2	33(8.78)
	Unknown	54	8	62(16.49)
Vaccination status	Fully vaccinated	100	4	104(27.66)
	Partially vaccinated	38	3	41(10.90)
	Up-to-date	167	6	173(46.01)
	Not vaccinated	26	5	31(8.24)
	Unknown	25	2	27(7.18)
Weight for age	Normal	323	13	336(89.36)
	Underweight	33	7	40(10.64)
Height for age	Normal	341	18	359(95.48)
	Stunting	15	2	17(4.52)
Weight for height	Normal	331	14	345(91.76)
	Wasting	25	6	31(8.24)

### Clinical characteristics and co-morbidities

Out of the 376 study participants, 304(80.85%) of children sought care within five days of an illness and 93(24.73%) of children had a history of acute respiratory tract infection. Among 376 study participants 222(59.04%) were febrile and about 252(67.02%) were hypoxic. The median temperature and oxygen saturation were 37.6 with IQR (36-38.1℃) and 87% IQR (82-90%) respectively. About 260(69.15%) developed danger signs, and the most frequent danger sign was vomiting 117(31.12%) followed by head nodding 48(12.76%). Among under-five children admitted with severe pneumonia, 191(50.80%) of them had co-morbidity. Hyperactive airway disease (childhood asthma) 102(27.13%) was the most frequent comorbidity followed by Heart disease 24(6.38%). As regards to the treatment options for severe pneumonia, 258(68.62%), 58(15.43%), and 60(15.96% were treated with Ceftriaxone, Crystalline penicillin, and Ampicillin & Gentamicin respectively (Table 3).

**Table 3:** Clinical characteristics of under-five children admitted with severe pneumonia in East Wallaga Zone public hospitals, Western Ethiopia, January 2017 to December 2022

Variables	Category	Survival status		Total (*n* = 376)
		Recovered	Censored	
		N*o*	N*o*	N*o* (%)
Duration prior to seeking care	≤ 5 days (Early presenters)	294	10	304(80.85)
	> 5 days (Late presenters)	62	10	72(19.15)
Past history of ARTI	Yes	87	6	93(24.73)
	No	269	14	283(75.27)
Oxygen saturation (Spo2)	< 90	239	13	252(67.02)
	≥ 90	117	7	124(32.98)
Temperature in ℃	s < 37.5 ℃	149	5	154(40.96)
	≥ 37.5 ℃	207	15	222 (59.04)
Presence of danger Sign	Yes	246	14	260(69.15)
	No	110	6	116(30.85)
Types of danger signs	Vomiting everything	112	5	117(31.12)
	Head nodding (15)	47	1	48(12.76)
	Grunting	46	1	47(12.50)
	Wheezing (35)	29	0	29(7.71)
	Chest indrawing	15	0	15(3.99)
	Abnormal body movement	5	4	9(2.39)
	Impaired consciousness	2	2	4(1.06)
Presence of Comorbidity	Yes	175	16	191(50.80)
	No	181	4	185(49.20)
Types of co-morbidity	HAAD	99	3	102(27.13)
	Heart disease	20	4	24(6.38)
	Malnutrition	19	4	23(6.12)
	AGE	21	1	22(5.85)
	Congenital anomaly	9	6	15(3.98)
	Anemia	11	1	12(3.21)
	Pertussis	9	0	9(2.39)
	Nephrotic syndrome	2	1	3(0.80)
	Measles	2	0	2(0.53)
Drug regimen	Crystalline penicillin	52	6	58(15.43)
	Ceftriaxone	249	9	258(68.62)
	Ampicillin & Gentamycin	55	5	60(15.96)

*Notes* AGE: Acute gastroenteritis

ARTI: Acute respiratory infection

HAAD: Hyperactive airway disease

### Treatment outcome and incidence rate of recovery from severe pneumonia

**Fig. 1:**
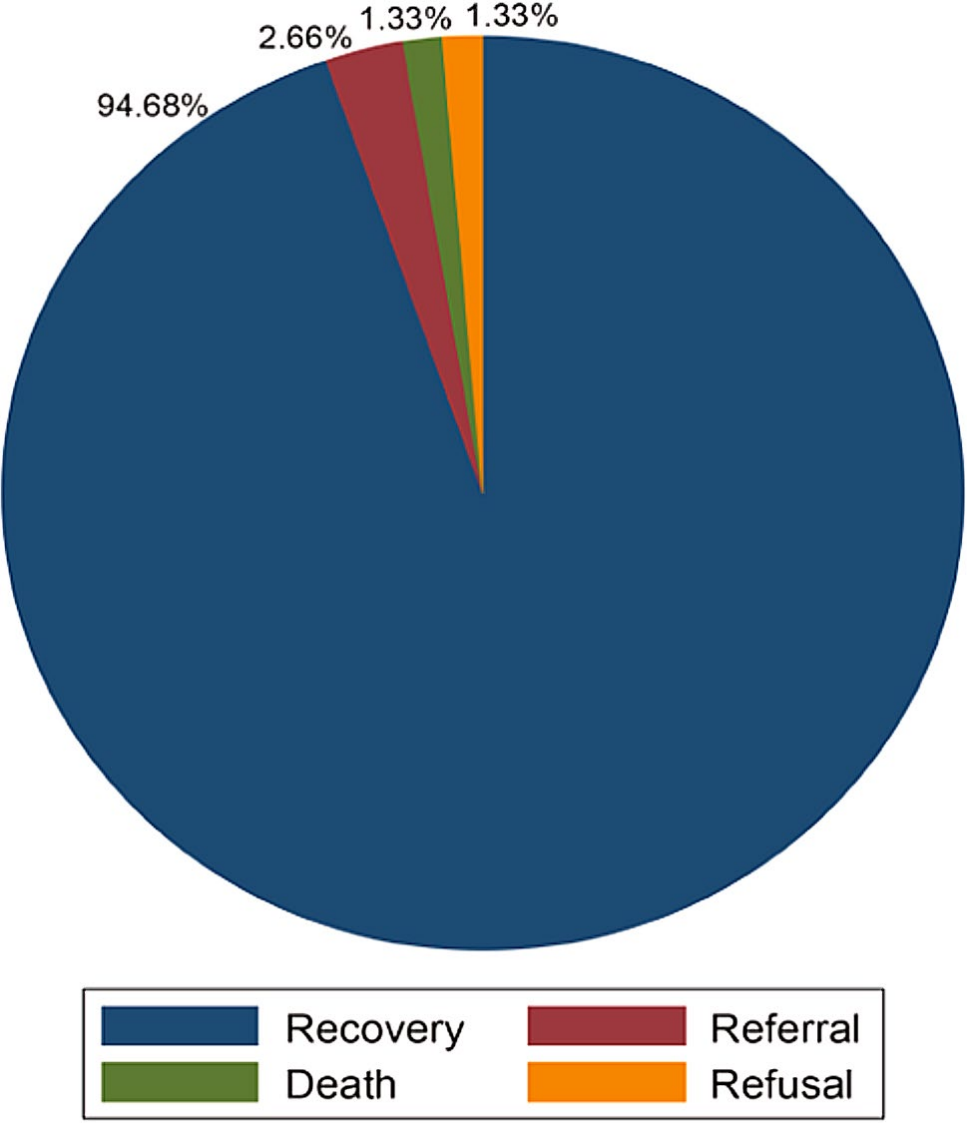
Treatment outcome among under-five children admitted with severe Pneumonia in East Wallaga zone public hospitals, Western Ethiopia, January 2017 to December 2022

The majority of the study participants 356(94.68%) recovered from their illness, while 5(1.33%) died, 10(2.66%) were referred to other health facilities for further treatment, and 5(1.33%) left against medical advice. During the follow-up time, a total of 1599 person-day risks were observed with a minimum and maximum follow-up time of 1 and 22 days, respectively. The overall incidence rate of recovery was 22.26 per 100 (95% CI: 20.07–24.70) person-days observations (Fig. 1).

### The median recovery time and comparisons of survival status between categories of predictors

Out of 376 total study participants admitted with severe pneumonia, about 356 (94.68%) developed an event (recovered). The overall median recovery time of under-five children admitted with severe pneumonia in east Wallaga zone public hospitals was 4 days with IQR of 3–5 days.

**Fig. 2:**
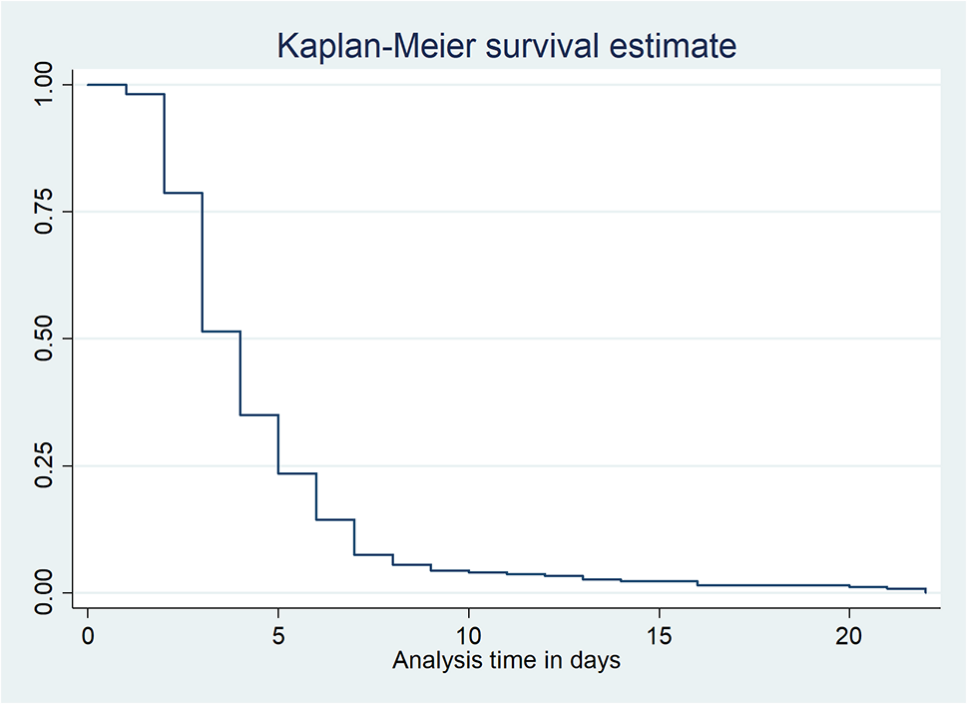
The overall Kaplan–Meier estimation of survival function from admission to recovery among under-five children admitted with severe pneumonia in East Wallaga zone public hospitals, Western Ethiopia, January 2017 to December 2022

A Kaplan-Meier estimation technique was used to see the estimate of survival time. The.

overall graph of Kaplan-Meier survival function depicted that the graphs decrease rapidly during the first 5 days showing most patients recovered from severe pneumonia during this time (Fig. 2).

**Fig. 3:**
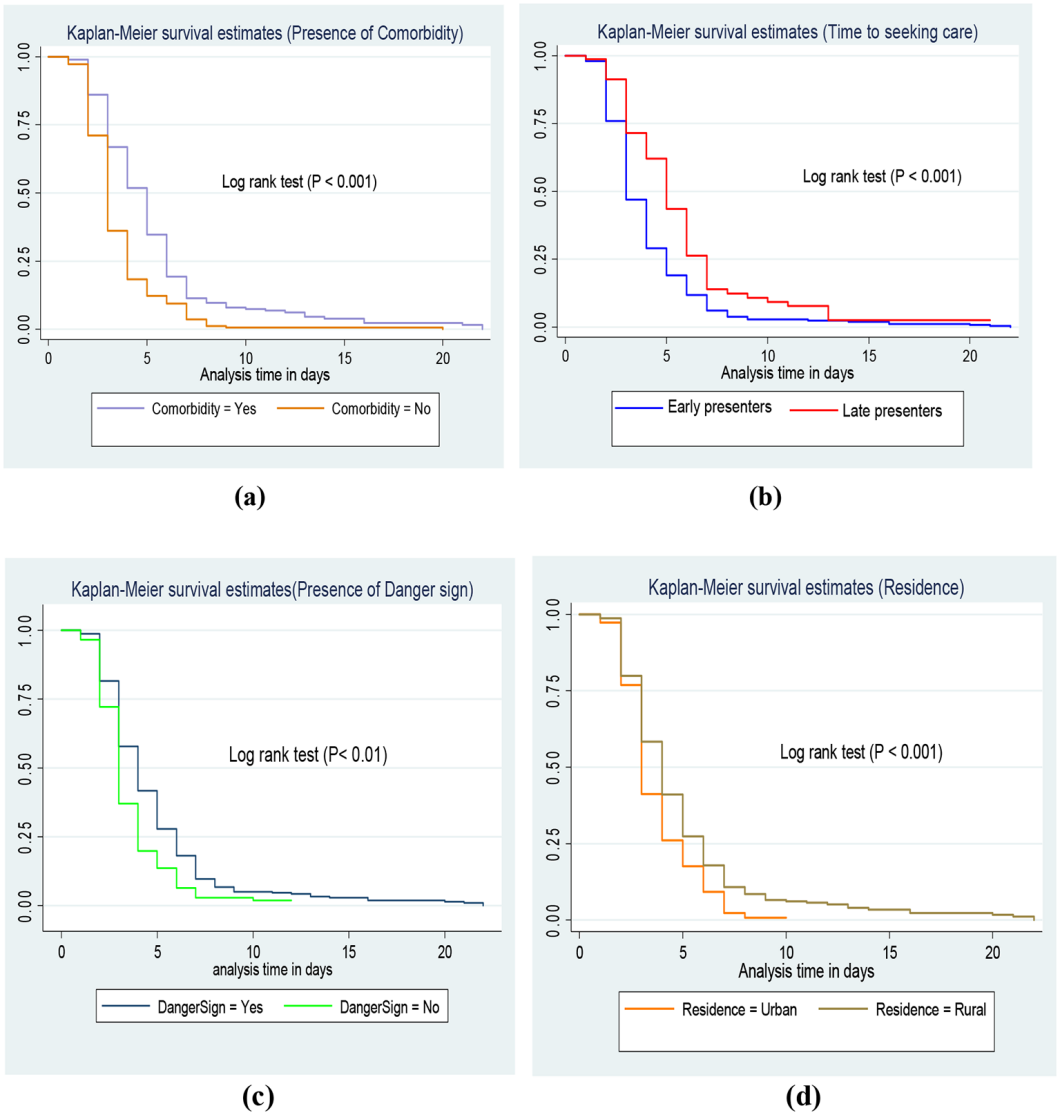
**(a)(b)(c)(d)** Kaplan-Meier survival estimate for time to recovery by categorical variables among under-five children admitted with severe pneumonia in East Wallaga zone public hospitals, Western Ethiopia, January 2017 to December 2022

A separate Kaplan-Meier curve was constructed to estimate the survival time based on different covariates to see the existence of differences in recovery rate between categories of individual covariates. From the Kaplan-Meier survival curve of individual covariates, there were understandable differences between groups of predictors. Based on the estimation, patients who had comorbidity, those children present lately to the hospital, had danger signs during admission, and children who reside in rural areas required a long time to recover from severe pneumonia compared to their counterparts respectively (Fig. 3).

To show the significance of survival difference, a log-rank test was computed at a 5% significance level. Accordingly, there was a significant difference in the survival status of patients concerning Age, Residence, Comorbidity, presence of danger signs at admission, Time prior to seeking care, and other baseline clinical characteristics (Table 4).

**Table 4:** Comparison of survival time to recovery among under-five children with severe pneumonia regarding different categories of predictors by using log-rank test in East Wallaga Zone public hospitals, Western Ethiopia, January 2017 to December 2022)

Variables	Category	Median recovery time in the day with IQR	Log-rank	*P*-value
		Point estimate (95%CI )	x2 value (df)	
Age-group	2–11months 12–35 months 36–59 months	4 (3-6) 3 (2-5) 3 (3-5)	9.06 (2)	0.0108
Residence	Urban Rural	3 (3-5) 4 (3-5)	12.07 (1)	0.0005
Weight for age	Normal Underweight	3 (3-5) 5 (3-6)	5.38 (1)	0.0204
Weight for height	Normal Wasting	3 (3-5) 6 (4-11)	13.14 (1)	0.0003
Temperature in ℃	< 37.5 ℃ ≥ 37.5 ℃	4 (3-5) 4 (3-6)	3.18 (1)	0.0747
Oxygen saturation	< 90 ≥ 90	4 (3-6) 3 (2-5)	3.33 (1)	0.0682
Duration prior to seeking care	≤ 5 days > 5 days	3 (3-5) 5 (3-7)	16.53 (1)	0.0000
Presence of danger Sign at admission	Yes No	4 (3-6) 3 (2-4)	16.50 (1)	0.0000
Comorbidity	Yes No	5 (3-6) 4 (3-5)	38.35 (1)	0.0000
Drug regimen	Crystalline penicillin Ceftriaxone Ampicillin & Gentamycin	3 (3-5) 3 (3-5) 4 (3-6)	3.93 (2)	0.1399

*Note* df: - degree of freedom IQR: Interquartile range

### Proportional hazard assumption test

In this study proportional hazard assumption test was checked graphically by log-log plot, and by using the Schoenfeld residual global test. Schoenfeld residuals proportional hazard assumption test for each covariate and the global test were used. If the P value is < 0.1 the assumption is rejected. In this study each covariant P-value is > 0.1 and the global test P-value is 0.6716, the result shows proportional hazard assumption was met.

### Model goodness-of-fit

**Fig. 4:**
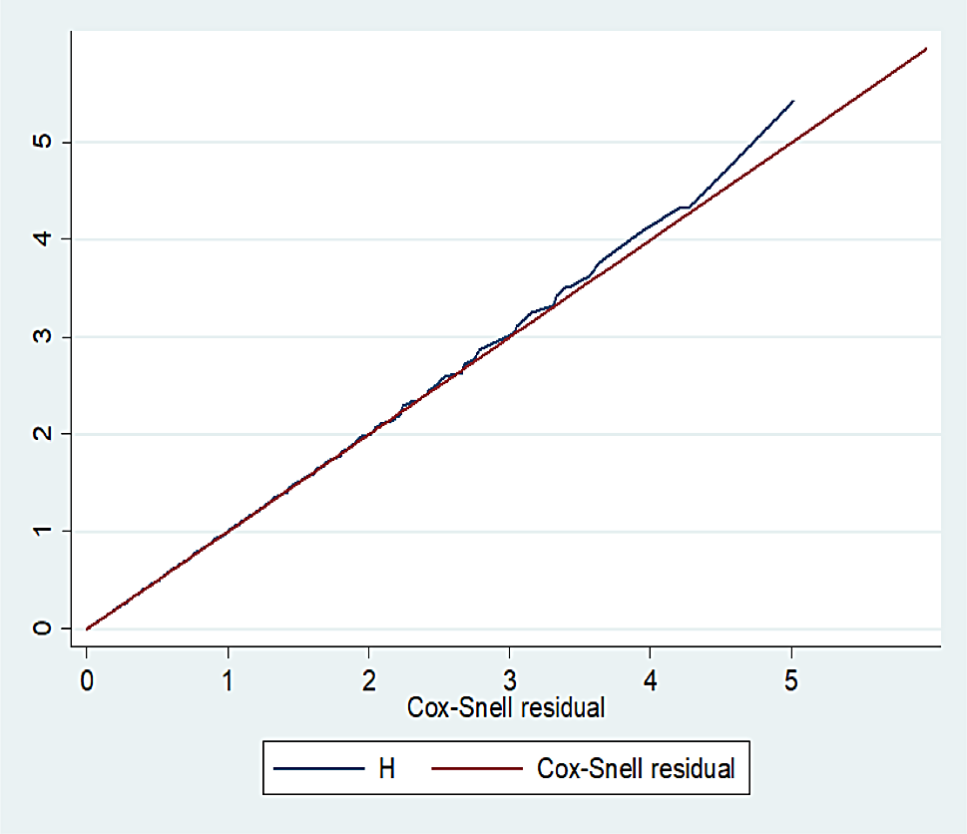
Cox Snell residual test for overall adequacy of the model among under-five children admitted with severe pneumonia in East Wallaga zone public hospitals, Western Ethiopia, January 2017 to December 2022

After fitting the multivariable Cox Proportional Hazard Model, the adequacy of model fitness was assessed by using Cox Snell residuals. Finally, the graph of the Nelson-Aalen cumulative hazard function and the Cox Snell residuals variable were compared to the hazard function to the diagonal line. The hazard function follows the 45-degree line, which approximately indicates that the model was well-fitted (Fig. 4).

### Predictors of recovery time from severe pneumonia

Covariates that had P- value < 0.25 in bivariable Cox regression analysis were selected for multivariable Cox regression analysis. Age, Residence, Weight for age, Weight for height, Comorbidity, Temperature, Oxygen saturation, Duration to seeking care, Drug regimen, and presence of danger signs at admission were selected for multivariable Cox regression at P-value ˂ 0.25. Finally, four predictors (Residence, comorbidity, duration prior to seeking care, and presence of danger sign at admission) were found to have a statistically significant association with recovery time during multivariable Cox proportional regression analysis.

According to the multivariable Cox regression analysis, the recovery time was delayed by 25% (AHR = 0.75; 95% CI (0.60–0.93)) among patients of rural residency as compared with its counterpart. Comorbidity was one of the predictors that affect the recovery time of children from severe pneumonia. The recovery time of children admitted with Comorbidity was 1.63 times longer (AHR = 1.63; 95% CI (1.31–2.04)) than that of children admitted without Comorbidity. This indicates that children admitted with Comorbidity had a longer time to recovery than children admitted without comorbidity. Regarding the duration of seeking care, children with severe pneumonia who came lately (˃5 days) to the health facility were 30% (AHR = 0.70; 95% CI (0.53–0.93)) delayed recovery time as compared to those who came early (≤ 5 days). The presence of danger signs at admission was another predictor that determines the recovery time of children with severe pneumonia. The recovery time of children admitted with danger signs was 1.46 times longer (AHR 1.46; 95% CI (1.15–1.83)) than that of their counterparts (Table 5).

**Table 5:** Multivariable Cox regression analysis of median recovery time and its predictors among under-five children admitted with severe pneumonia in East Wallaga Zone public hospitals, Western Ethiopia, January 2017 to December 2022

Variables	Category	Survival status		CHR(95% CI)	AHR(95% CI)	P-value
		Recovered	Censored			
Age group	2–11months	175	8	1	1	
	12–35 months	127	7	1.32(1.05–1.66)	1.26(1.01–1.57)	0.051
	36–59 months	54	5	1.28(0.94–1.74)	1.19(0.85–1.67)	0.110
Residence	Urban	143	5	1	1	
	Rural	213	15	0.72(0.59–0.90)	0.75(0.60–0.93)	0.010*
WFA	Normal	323	13	1	1	
	Underweight	33	7	0.70(0.49–1.01)	1.28(0.50–1.03)	0.913
WFH	Normal	331	14	1	1	
	Wasting	25	6	0.53(0.37–0.80)	0.98(0.44–2.27)	0.105
Time prior to seeking care	≤ 5 days (Early presenters)	294	10	1	1	
	> 5 days (Late presenters)	62	10	0.62(0.47–0.82)	0.70(0.53–0.93)	0.014*
Danger Sign	No	110	6	1	1	
	Yes	246	14	1.48(1.18–1.86)	1.46(1.15–1.83)	0.001**
Temperature in ℃	< 37.5 ℃	149	5	1	1	
	≥ 37.5 ℃	207	15	0.84(0.68–1.05)	0.835(0.67–1.05)	0.159
Oxygen saturation	≥ 90	239	13	1	1	
	< 90	117	7	1.19(0.95–1.48)	1.15(0.91–1.44)	0.384
Co-morbidity	No	181	4	1	1	
	Yes	175	16	1.74(1.41–2.16)	1.63(1.31–2.04)	< 0.001**
Drug regimen	Crystalline penicillin	52	6	1	1	
	Ceftriaxone	249	9	0.89(0.66–1.20)	0.98(0.72–1.33)	0.896
	Ampicillin & Gentamycin	55	5	0.73(0.50–1.07)	0.85(0.55–1.28)	0.358

*Notes* AHR: Adjusted Hazard Ratio; CHR: Crude Hazard Ratio

* p-value < 0.05; ** p-value ≤ 0.001

1: Reference WFA: Weight for age; WFH: Weight for height

## Discussion

This study intended to determine the time to recovery and its predictors among under-five children admitted with severe pneumonia in East Wallaga zone public hospitals, western Ethiopia. This study pointed out that the median time to recovery from severe pneumonia was 4 days (95% CI: 3–5). Different predictors were contributing to the recovery time of children admitted with severe pneumonia. Residence, comorbidity, presence of danger signs at admission, and duration prior to seeking care were found to be predictors of time to recovery from severe pneumonia.

The finding of this study is in line with the study conducted in Debre Markos referral hospital [21], Ayder Comprehensive Specialized hospital [20], and Gonder, Ethiopia [17] in which recovery time lasted for 4 days. This might be due to the relative similarity in care and treatment given to the patients in the study areas This short.

duration of the hospital stay could also reflect the good quality of.

the care and adherence of the service providers to the standard.

treatment. However, this duration is lower than study findings from the rural health center of Gambia which reported the median time of recovery (5 days) [15] and the duration at Mulago Hospital Uganda (7 days) [28], and higher than the study done in Vanderbilt university hospital, USA (2 days) [29] and Nepal (2 days) [16]. This discrepancy might be due to the time difference in which the studies were conducted. Another possible reason for this inconsistency might be related to differences in treatment and caring practices, sample size, healthcare settings, and other socioeconomic factors between areas where the studies were conducted.

This study showed that residency was a significant predictor of recovery time of under-five children admitted with severe pneumonia; children who were rural residents had recovery dalliance as compared to their counterparts. This finding is in line with a study conducted in Central and North Gondar zone public hospitals, in Northeast Ethiopia, that rural residency prolongs the length of recovery [17]. However, this finding contradicts a study conducted in Egypt, which revealed that being an urban resident was the risk of a long recovery time from pneumonia [30]. This result suggested that, most of the time people living in rural Ethiopia are economically poor and they may not afford the cost of the treatment. Due to that, they do not take their sick children early to health facilities and this dalliance worsens the severity of the disease, which consequences slow the recovery time [31]. Furthermore, undernutrition is more common in Ethiopia’s rural areas than in its urban, and it is clear that this causes immunological suppression, which lengthens the recovery time [32].

Duration prior to seeking care was a significant predictor for the recovery time of children with severe pneumonia. Those children who presented to the hospital late (after five days of illness) had longer recovery time as compared with those children who presented early (before five days of illness). This finding is supported by similar studies done in Debre Markos referral hospital [21], Gondor [17], and Gambia [15]. The possible reason could be related to the time to seek treatment that the progression of the disease increases as children are late to seek treatment which leads to a long period of hospitalization and poor treatment outcomes. Similarly, as a disease progresses, the amount of time it takes to recover increases as well.

The presence of co-morbidity was the other important predictor which was significantly associated with recovery time from severe pneumonia. In this study, HAAD, Heart disease, AGE, pertussis, Congenital anomaly, Measles, and other diseases were considered as co-morbid diseases with severe pneumonia. Children who were admitted to hospitals with co-morbidity took a longer time to recover compared to their counterparts who were free of co‑morbidities. This is due to the fact that when children acquire different illnesses at a time; their immune system significantly decreases, which prolongs the length of hospital stay and delays the time of recovery. This finding is in line with other different studies done at the University of Gondar Comprehensive Specialized Hospital [19], Debre Markos Referral Hospital [21], Gambia [15], and Nepal [16].

The current study suggested that those children admitted with severe pneumonia and who had danger signs at admission had a lower recovery rate than those admitted without danger signs. This finding was consistent with other similar studies conducted in Gambia, Nepal, and Ethiopia [15, 16, 21]. This indicates that those children admitted without danger had a shorter recovery time than their counterparts. This could be due to the reason that the effect of severity of the disease. As the disease becomes severe, the required time to recovery from the disease becomes too long due to the pathophysiologic state of the disease [33].

### Conclusion and recommendations

In general, the median time to recovery from severe pneumonia as well as possible predictors was assessed in this study. The median time to.

recovery from severe pneumonia was short according to the.

US Centers for Disease Control and Prevention. Rural residence, presence of danger signs at admission, late presenters for seeking care, and presence of co-morbidity were predictors that can increase the length of hospitalization. Hence, due attention have to be given in increasing the community’s health-seeking behavior to visit health facility early and especial attention should be given for children with danger signs and comorbidity. It is also recommended that for better knowledge, future researchers need to conduct a prospective cohort study that includes other variables such as parental and socio-economic characteristics, as well as caregivers’ perceptions towards severe pneumonia.

### Electronic supplementary material

Below is the link to the electronic supplementary material.

Supplementary Material 1

## Data Availability

The data used to support the findings of this study is available from the corresponding author upon request.

## Abbreviations

AGE: Acute Gastroenteritis
AHR: Adjusted Hazard Ratio
AIDS: Acquired Immuno Deficiency Syndrome
ARTI: Acute Respiratory Tract Infection
CHD: Congenital Heart Disease
CHR: Crude Hazard Ratio
DAMA: Discharge Against Medical Advise
DM: Diabetes Mellitus
HAAD: Hyper Active Airway Disease
HIB: Haemophilus Influenzae Type b
HIV: Human Immuno Virus
HMIS: Health Management Information System
IMCI: Integrated Management of Childhood Illness
IQR: Inter Quartile Range
ORS: Oral Rehydration Salt
PCV: Pneumococcal Vaccine
SAM: Severe Acute Malnutrition
US: United State
VIF: Variance Inflation Factor
WHO: World Health Organization
