# Supplementary material for: Time to recovery and its predictors among under-five children admitted with severe pneumonia in East Wallaga Zone public hospitals, western Ethiopia, 2023; a retrospective cohort study
Source: BMC Pediatr. 2024 Jul 18;24:459. doi: 10.1186/s12887-024-04937-2 (PMC11256476; doi:10.1186/s12887-024-04937-2)
Supplement: Supplementary file 1 — Supplementary Material 1 [file 12887_2024_4937_MOESM1_ESM.docx]

| **Table** :- Proportional hazard assumption test by Schoenfeld residuals for each predictor and global test among under-five children admitted with severe pneumonia in East Wallaga zone public hospitals, Western Ethiopia, January 2017 to December 2022. | | | |
| --- | --- | --- | --- |
| **Predictors** | **Chi2** | **Df** | **Prob>chi2** |
| Residence | 0.03 | 1 | 0.8571 |
| Comorbidity | 1.60 | 1 | 0.2054 |
| Age group | 0.02 | 1 | 0.8786 |
| Temperature in ℃ | 0.61 | 1 | 0.4341 |
| Oxygen saturation | 0.02 | 1 | 0.8745 |
| Duration prior to seeking care | 2.42 | 1 | 0.1200 |
| Danger Sign | 0.51 | 1 | 0.4739 |
| Weight for age | 0.22 | 1 | 0.6387 |
| Weight for height | 1.81 | 1 | 0.1786 |
| Drug regimen | 1.05 | 1 | 0.3060 |
| **Global test** | **7.56** | **10** | **0.6716** |
